# Learning the role together: experiences of using peer learning in the introductory training of newly employed nurses in the ambulance service

**DOI:** 10.1186/s12909-026-08992-4

**Published:** 2026-03-12

**Authors:** Klara Jepsen, Ann-Charlotte Falk, Annika Alm, Jonas Wihlborg, Veronica Lindström

**Affiliations:** 1 Department of Neurobiology, Care Sciences and Society, Karolinska Institutet, Stockholm, Sweden; 2 Samariten Ambulance AB, Stockholm, Sweden; 3 Department for Health Promoting Science, Sophiahemmet University, Stockholm, Sweden; 4 School of Health and Welfare, Dalarna University, Falun, Sweden; 5 Department of Nursing, Division of Ambulance Service, Region Västerbotten, Umeå University, Umeå, Sweden

**Keywords:** Ambulance service, EMS, Introduction, Newly employed, Nurses, Paramedic, Ambulance personnel, Peer learning, Teams

## Abstract

**Background:**

Working in the ambulance service is complex and unpredictable, requiring newly employed nurses to rapidly assume autonomous clinical responsibility. Introductory training often relies on traditional supervision models that may not fully support transition into practice. Peer learning (PL) has been shown to promote collaboration, confidence, and professional development in educational settings; however, its use during workplace introduction in ambulance services remains largely unexplored.

**Aim:**

To describe the use of PL in the introductory training of newly employed nurses in the ambulance service and how it was experienced by newly employed nurses and clinical supervisors.

**Design:**

A qualitative descriptive study with an inductive thematic analysis approach was used.

**Methods:**

Data were collected through individual and focus-group interviews and analysed using reflective thematic analysis as outlined by Braun and Clarke. Four clinical supervisors and ten newly employed nurses who had PL in their introductory training participated in the study. The study follows the COREQ checklist for reporting qualitative research.

**Results:**

This study explored the experiences of newly employed nurses and clinical supervisors of PL during the ambulance service’s introductory training. Three main themes were identified: *becoming an independent professional*, *safe space for shared learning*, and *a balancing act as a supervisor*. PL supported the gradual transition into autonomous practice, facilitated emotional and professional support through peer collaboration, enhanced role clarity, and emphasized the importance of supervisors in fostering independence while providing accessible guidance. Across all themes, feedback and reflection were central to learning, helping nurses make sense of their experiences, gain confidence, and adapt to the demands of the ambulance context. PL was seen as a valuable, structured learning phase that promotes mutual learning and professional development for new nurses.

**Conclusions:**

PL represents a valuable structured learning phase within a structured onboarding programme for newly employed nurses in the ambulance service. By combining PL with facilitated supervision and reflection, PL may support a safe and sustainable transition into practice in complex clinical environments.

## Introduction

 In emergency medical services (EMS), ambulance services play a critical role in delivering safe, high-quality care to individuals outside hospitals [1–5]. Newly employed nurses entering this clinical and patient-facing setting are expected to act as competent and autonomous professionals, making rapid decisions in unpredictable clinical environments while managing diverse patient presentations after their introduction training [2, 6]. Such expectations placed early in employment risk creating a mismatch between expectations placed on newly employed nurses and their actual prehospital emergency care-specific knowledge, underscoring the need for structured and supportive onboarding interventions. Despite the importance of effective onboarding, structured forms of peer learning (PL) have rarely been used in introductory training within the ambulance service. This study, therefore, aimed to describe the use of PL in the introductory training of newly employed nurses in the ambulance service and how it was experienced by newly employed nurses and clinical supervisors.

PL is widely used in undergraduate and advanced nursing education and has been shown to support professional development, collaborative skills, and self-efficacy [7–14]. It has been shown that working with and supporting peers reduces anxiety and stress and encourages shared problem-solving and reflection [15–20].

However, research on PL in the ambulance service, particularly during introductory training for newly employed nurses and involving clinical supervisors, is limited.

### Background

The ambulance service provides prehospital emergency care to individuals during accidents or acute illness outside hospitals [5, 21]. Globally, ambulance services differ in professional composition and onboarding structures. In many countries, prehospital emergency care is delivered primarily by paramedics who complete structured internships following undergraduate education, during which peer collaboration is integral to onboarding. In contrast, in some European countries, including Sweden, registered nurses (RNs) constitute core members of ambulance teams [22]. These nurses commonly enter ambulance services with general nursing education and limited formal prehospital emergency care training, unless they have completed a one-year additional specialist education, yet they are expected to perform with a high degree of autonomy in complex clinical situations [2, 23, 24]. Consequently, newly employed RNs enter the ambulance service with limited prehospital emergency care-specific experience and knowledge, highlighting the importance of structured introductory training.

It is known that starting work in the ambulance service can be overwhelming [4, 6] as the newly employed nurses, after introductory training, are expected to manage patient care independently, use context-specific equipment, and meet organizational expectations [2]. Adjusting to a new environment may lead to stress and vulnerability [24, 25], while structured onboarding and experienced clinical supervisors can ease this transition and help nurses gain confidence [2].

Nurses play a central role in care coordination and teamwork, both of which are crucial in high-pressure situations [26, 27]. In addition, the ambulance teams often consist of professionals with varying educational and experiential backgrounds, and team members may or may not have worked together previously [28].

In the Swedish ambulance service context examined in this study, a common supervision model in introductory training is the master–apprentice approach [29], in which the novice learns from the experienced supervisor by observing and gradually taking on tasks independently. While this model enables knowledge transfer and socialization into workplace culture, it also risks passive learning and alignment with the supervisor’s worldview rather than independent critical thinking [29, 30].

In response to these limitations, alternative ways of organising learning that promote active engagement and shared responsibility have been suggested. One example is PL, which is grounded in social learning theory [28]. In PL, learners work together, sharing responsibility for patient care and learning, engaging in collaboration, feedback, and reflection. Such interaction has been shown to deepen engagement and support the development of evaluative judgement [31, 32]. In this study, the PL is used consistently. Activities such as feedback, teaching, and peer interaction are understood as integral processes within PL rather than as separate learning concepts.

Previous studies from nursing education demonstrate that PL can increase clinical knowledge, self-efficacy, and confidence, while reducing anxiety and stress among learners [8–13]. PL contributes to the development of knowledge, understanding, and confidence, and both clinical supervisors and peers play important roles in supporting self-efficacy and influencing how individuals approach tasks and cope with challenges [14]. Despite these documented benefits, PL used in introductory training within nurse-led ambulance services remains underexplored. Conte et al. [16] described interprofessional learning for undergraduate students in prehospital settings, showing that ambulance services can support learning in unpredictable care scenarios. However, variability in supervision quality and a need for enhanced supervisor training were identified [16]. Research further suggests that peers’ diverse knowledge and experience enrich learning outcomes in complex professional fields by enabling learners to draw on multiple perspectives and clinical experience [33]. Nevertheless, when supported by clear frameworks and engaged leadership, PL remains a promising form of structured learning for introductory training [17, 19].

Being a clinical supervisor in the ambulance service is complex, involving socialising, coaching, and supporting newly employed personnel. Clinical supervisors must balance patient safety and care with supervision and adopt multiple roles: facilitator, stimulator, teacher, and team player [18, 20]. Described by Dyar et al. [20], the *teacher* role reflects a traditional, instructor-centred approach in which supervisors primarily provide information and direction. As *facilitators*, supervisors focus on creating opportunities for students to work together and supporting their reflective engagement. In the *stimulator* role, supervisors actively encourage students to take initiative, ask questions, and challenge one another to deepen learning. The *team player* role represents the broadest understanding, in which supervision is viewed as a shared responsibility across the clinical team and peer learning is integrated into everyday collaborative practice. Together, these roles illustrate a progression from directive teaching to a more participatory, team-based learning environment [20]. To our knowledge, no studies have described PL as part of introductory training for newly employed nurses in ambulance services, nor explored clinical supervisors’ experiences of using PL in this context. Accordingly, this study addressed the following research question: How do newly employed nurses and their clinical supervisors experience PL during introductory training in the ambulance service?

## The study aim

This study aimed to describe the use of PL in the introductory training of newly employed nurses in the ambulance service and how it was experienced by newly employed nurses and clinical supervisors.

## Methods

### Design

A qualitative design was used to describe the experiences of newly employed nurses and clinical supervisors of PL during the introductory training in the ambulance service in Stockholm, Sweden. Thematic analysis, according to Braun and Clarke [34], was used to analyse data collected from focus groups and individual interviews with ten newly employed nurses and four clinical supervisors. The study presentation conforms to the COREQ checklist for reporting qualitative research [35]. This study was funded by Samariten Ambulans AB.

### Study setting

The study was conducted in Stockholm, Sweden, a region with around 2.2 million residents. During the study period, two contracted organizations operated ambulance services under the regional county council, and approximately 22 RNs and 14 SNs began introductory training for work in the services. At Samariten Ambulans AB, the eight-week introductory training includes three weeks of theory, one week of driving, and four weeks of supervised clinical training. While there are no formal requirements for clinical supervisors, the expectation is that they should be SNs with at least one year of ambulance experience.

In Sweden, to qualify as an RN in the ambulance service, nurses must hold at least a bachelor’s degree and one year of relevant experience in an emergency care setting, such as an emergency department. RNs in Sweden complete a three-year undergraduate education with a nationally regulated structure and learning outcomes defined by the Swedish Council for Higher Education [36]. In some regions, including Stockholm, one ambulance team member must be an SN [37]. Becoming an SN requires an additional year of advanced postgraduate studies, leading to a master’s degree [36]. Most SNs in ambulance services specialise in ambulance, intensive, or anaesthesia care [38]. SNs may work alongside an RN or an Emergency Medical Technician (EMT) with basic life support skills [39]. In this study, the term newly employed nurses includes both RNs and SNs.

#### Peer learning in the ambulance service

In this study, PL was initially understood as a structured instructional method intentionally introduced into the introductory training programme. During the design phase, PL referred to nurses with similar professional backgrounds who were not acting as formal instructors but supported each other’s learning through teaching and collaboration. Consequently, the introductory training programme was organised to actively foster PL through shared clinical responsibility, structured reflection, and supervisor-led dialogue. PL did not develop informally but was intentionally organised through predefined peer pairing, planned reflective activities, and active supervisory guidance during clinical training.

In 2020, a pilot project was conducted to explore the feasibility of implementing PL in the introductory training of newly employed nurses in the ambulance service. Two PL teams were established, each comprising two newly employed nurses who worked together during clinical training. Four clinical supervisors were selected for the pilot based on their pedagogical interests and prior experience in clinical supervision. All supervisors were specialist nurses (SNs) with extensive ambulance service backgrounds and participated in a half-day theoretical PL session to develop a shared understanding of PL principles and supervisory roles. The pilot project demonstrated that PL was feasible in terms of logistics, ambulance weight limits, patient safety, and learning conditions. Based on these findings, PL was implemented in 2022 as part of the regular introductory training programme. Before starting PL, newly employed nurses received written information and attended a preparatory half-day session introducing the principles of PL, including the use of Gibbs’ reflective cycle [40] as a structured framework for reflection. PL was tailored to fit the ambulance service context, where patient safety and clinical demands always take priority. During the PL period, two newly employed nurses worked together as a peer pair in the ambulance, with clinical supervisors present throughout the clinical shifts. Supervisors retained overall responsibility for patient safety and, when necessary, participated in patient care, but primarily took on a facilitating role to support PL between nurses.

The peer pairs were encouraged to share responsibility for patient assessment and care, alternate leading roles, and discuss clinical decisions during and after patient encounters. Supervisors supported learning by posing reflective questions and encouraging dialogue between peers rather than providing direct instruction. In situations involving critically ill patients, supervisors occasionally assumed a more directive role, and learning temporarily shifted towards observation, followed by structured reflection during debriefings.

Reflection was integrated throughout the shifts and typically took place after patient encounters and during structured debriefings at the end of each shift. During these sessions, clinical supervisors facilitated group reflection by encouraging the newly employed nurses to discuss their clinical reasoning, compare experiences, and consider alternative actions.

PL was further supported by supervised group reflection, in which the nurses were encouraged to respond to each other’s reflections rather than address the supervisor directly. During the PL period, the nurses were also encouraged to identify individual and shared knowledge gaps and to prepare for upcoming shifts accordingly.

**Fig. 1:**
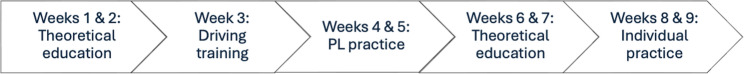
A nine-week introductory training programme for newly employed nurses in the ambulance service

Clinical supervisors had the same qualifications as in the pilot project, with two additional supervisors included after implementation. All supervisors completed the same preparatory PL session as the nurses and reflected on how PL could be applied consistently in clinical practice. Teams were pre-assigned and, when possible, consisted of one SN and one RN; when SNs were unavailable, two RNs formed the peer pair. The overall introductory training followed a structured onboarding process, during which PL established a clearly defined learning phase in Weeks 4–5 (Fig. 1).

After the two-week PL period, the introductory training continued with theoretical education followed by individual clinical pracatice alongside a regular ambulance team. Reflection and supervision continued during this phase, although without the structured peer pairing characteristic of PL. Thus, clinical supervisors mainly acted as facilitators of PL rather than traditional instructors. Learning during the PL phase, therefore, occurred through interactive mechanisms, including peer collaboration, supervisor facilitation, and observation when necessary for patient safety; however, PL structured how experiences were discussed, reflected upon, and turned into learning.

### Participants

#### Newly employed nurses

Sixteen nurses assigned to PL during their introductory training in the ambulance service between 2022 and 2024 received an email with study information and an invitation to participate. A reminder email was sent two weeks later. Ten newly employed RNs agreed to participate, including three SNs. Eight participants took part in focus-group interviews, while two participated in individual interviews due to scheduling constraints. The participants were not asked about their previous experience with PL. Participant characteristics are presented in Table 1.

**Table 1:** Newly employed nurses (*n* = 10) participating in interviews

Age (years)	
Range	26–49
Mean/median	34/30.5
Gender	
Female	9 (90%)
Male	1 (10%)
Degree	
Registered nurse	7 (70%)
Specialist nurse	3 (30%)
Work experience as a nurse in the healthcare service (years)	
1–5	7 (70%)
6–10	1 (10%)
11–15	2 (20%)

#### Clinical supervisors

Four of six eligible clinical supervisors were personally approached and provided with information about the study. Of these, three participated in focus-group interviews and one in an individual interview due to scheduling constraints. Clinical supervisors’ characteristics are presented in Table 2.

**Table 2:** Clinical supervisors (*n* = 4) participating in interviews

Age (years)	
Range	34–57
Mean/median	45.5/45.5
Gender	
Female	2 (50%)
Male	2 (50%)
Degree	
Specialist nurse	4 (100%)
Work experience in the ambulance service (years)	
6–10	3 (75%)
>16	1 (25%)

### Data collection

Four focus-group interviews were conducted: three with newly employed nurses, each with 2–4 participants, and one with clinical supervisors (*n* = 3). In addition to the focus-group interviews, three individual interviews were conducted with participants who could not attend the scheduled focus-group interviews, one with a clinical supervisor and two with newly employed nurses.

Interview guides, inspired by Pålsson [7], were developed by the first author and reviewed by the co-authors: one for newly employed nurses and one for clinical supervisors. The interview guides each consisted of eleven main questions, which differed slightly between the guide for newly employed nurses (Table 3) and the guide for clinical supervisors (Table 4). In addition, follow-up questions were used to explore and clarify responses as needed, yielding deeper insights into participants’ experiences and perspectives. The interviews were conducted via Zoom and recorded using Zoom’s recording function. The interviews were transcribed verbatim. The interviews lasted 18–54 min (median 39 min). Data were collected in 2024 by two of the authors (KJ and VL).

**Table 3:** Interview guide for the newly employed nurses

Questions
1. Can you tell us about your experiences with peer learning?
2. How did you deal with challenges? (from the organizational/supervisor and patient perspectives)
3. Did you have any peer support?
4. How did the supervisors facilitate your learning/professional and personal development?
5. How did you handle conflicts/competition among you as peers?
6. How did you get feedback (individual/pair)?
7. Did you start from any structured working methods/models?
8. How were you introduced to peer learning?
9. How can the concept of peer learning be developed further?
10. Describe a peer learning event more specifically.
11. Is there anything else you can think of or want to add?

**Table 4:** Interview guide for clinical supervisors

Questions
1. Can you tell us about your experiences with peer learning?
2. How did you deal with challenges? (from the organizational/supervisor and patient perspectives)
3. Did you have any peer support?
4. How did you facilitate the learning/professional and personal development of the newly employed nurses?
5. How did you handle conflicts/competition among the newly employed nurses?
6. How did you give feedback (individual/pair)?
7. Did you start from any structured working methods/models?
8. How were you introduced to peer learning?
9. How can the concept of peer learning be developed further?
10. Describe a peer learning event more specifically.
11. Is there anything else you can think of or want to add?

### Data analysis

The four focus groups and three individual interviews were analysed using an inductive approach, employing reflexive thematic analysis as described by Braun and Clarke [41]. As the phenomenon has not been studied to any great extent and we accordingly lack general principles, an inductive approach was used [41]. The method involves analysing the data in five steps to identify themes, followed by step six, which consists of writing up the results. Step one: The researcher (KJ) familiarized herself with the data. Step two: KJ developed initial codes using Atlas.fi software and included all the data. Step three: All codes were grouped into preliminary sub-themes by sorting the codes with similar content, and the sub-themes were colour-coded to highlight their structure. At this stage of the analysis, KJ and AA received the material and discussed the sorting and naming of the sub-themes, reaching consensus on the first steps of the analysis. Step four: During steps three and four, the KK, AA, and VL compared the codes, discussed similarities and differences, and reorganised them into preliminary categories. Step five: The authors reviewed the categories and identified eight sub-themes that represented patterns across the dataset. These sub-themes were subsequently grouped into three themes and continuously reviewed against the transcribed data to ensure consistency and accuracy. All stages of the analysis were discussed among the authors, and necessary refinements were made in accordance with Braun and Clarke [41]. Table 5 gives examples of the analysis process. Step six: Included the presentation of the results.

**Table 5:** Example of the analysis process

Text	Code	Sub-theme	Theme
“What I thought was so good about peer learning was that you had a person at the same level as yourself, even if you had different experiences, you could still feel in the situation that you could ‘bond’ with someone at the same level.” (2:1)	Ability to bond through shared experiences	Mutual understanding through shared experiences	The power of peer teams

### Rigour and reflexivity

Data were collected by two authors (KJ and VL). To ensure consistency and coherence in the data collection process, efforts were made to maintain a consistent approach among the interviewers before and after the interviews. KJ and AA led each process step during the analysis phase, while the remaining authors reviewed and validated the analyses. When discrepancies arose, discussions among all authors were held to reach a consensus. The section “Strengths and limitations of the work” provides a more detailed discussion of the study’s limitations.

Reflexivity played an essential role in interpreting and understanding the data. The preconceptions of three authors (i.e., KJ, VL, and JW) regarding the study context, arising from their extensive experience working as SNs in ambulance services, could introduce bias during the study design, data collection, and analysis phases, influencing the analytical process. However, these preconceptions of the context could also be considered strengths, as they facilitated nuanced insights into the findings. In contrast, two authors (i.e., AA and ACF) had no experience in ambulance services, giving them an external perspective that helped balance the interpretation and analysis.

## Results

**Fig. 2:**
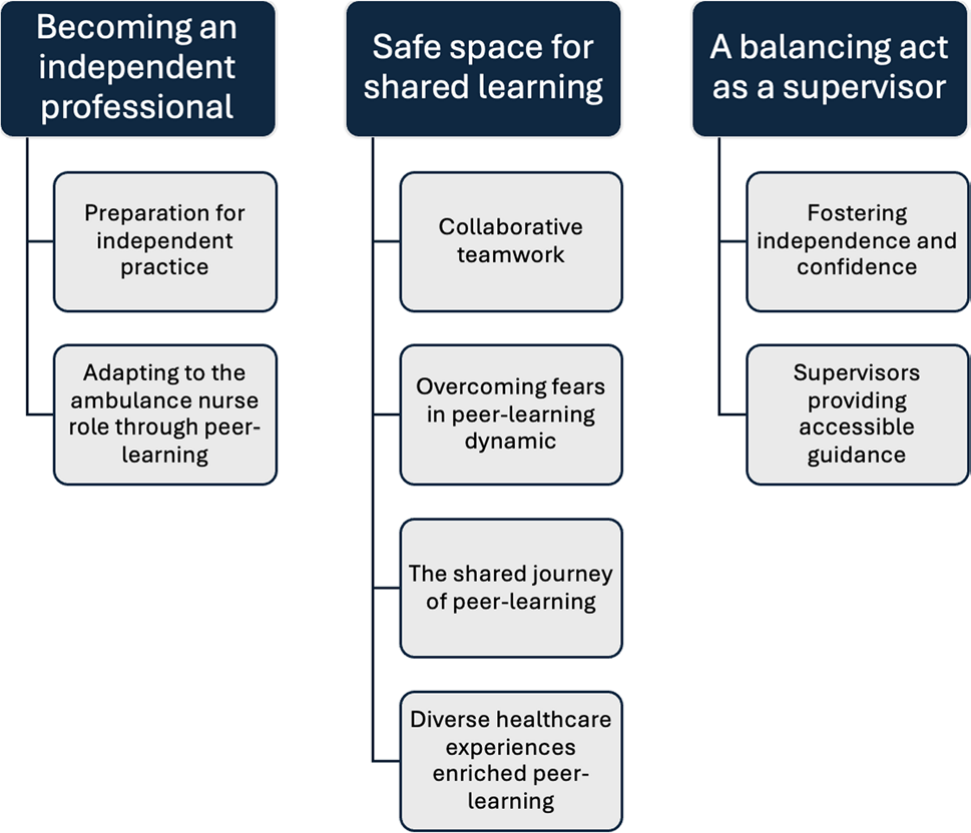
Overview of themes and sub-themes

Three themes resulted from the analysis, reflecting the experiences and perspectives of newly employed nurses and their clinical supervisors. The *first theme*, becoming an independent professional, describes how PL facilitated a gradual transition into autonomous practice. PL enabled newly employed nurses to build confidence in clinical decision-making through a progression that combined hands-on experience with continuous, dialogical support from clinical supervisors. The *second theme*, safe space for shared learning, emphasizes how peer collaboration fosters emotional reassurance, mutual learning, and a sense of belonging. This shared learning was created within PL through scheduled peer reflection and collective discussion of clinical experiences, rather than occurring informally. However, according to the nurses and clinical supervisors, group dynamics were challenging at times. Finally, the *third theme*, a balancing act as a clinical supervisor, explores how the clinical supervisors played a key role in creating a learning environment that balanced support with independence. Within PL, the supervisor role extended beyond clinical oversight to include considered pedagogical facilitation, where guidance, role modelling, and structured reflection were used to support professional development. Their ability to create space for reflection and to progressively transfer responsibility was essential to the newly employed nurses’ development and constitutes a defining feature of PL, distinct from traditional introduction training. An overview of the results, including the identified themes and sub-themes, is presented in Fig. 2.

Across all three themes, feedback and reflection were central to PL. These elements were integrated into the PL period through planned reflection sessions, guided feedback, and shared learning goals, distinguishing PL from more traditional introductory training, where learning often occurs implicitly through exposure to practice. These elements enabled the nurses to make sense of their experiences, deepen their understanding, and build the confidence needed to assume their professional roles in a demanding and unpredictable environment. Furthermore, the results indicate that the newly employed nurses experienced learning and professional development during their introduction to the ambulance service and attributed this development to the structured, longitudinal, and pedagogically guided nature of PL, rather than to workplace exposure alone. The participants described PL as a good, structured learning model that made learning visible, discussable, and assessable over time, in contrast to previous experiences of more ad hoc introductions.

### Becoming an independent professional

#### Preparation for independent practice

Both newly employed nurses and clinical supervisors described and discussed the use of PL to enable nurses to gradually transition to independent practice. PL allowed the nurses to build confidence in their decision-making, better understand the practical aspects of their work, and develop essential skills required for an autonomous role as nurses in the ambulance service:

After doing the peer learning, you feel like, okay, now we’re going out alone, but you still know what it’s about and how it works. It doesn’t feel like such a big step compared with being thrown in alone back there, with no one to discuss things with. (Newly employed nurse, 3:20)

In addition to learning clinical and technical skills, PL reinforced the nurses’ emotional readiness for independent practice. They felt confirmed by their peers and supervisors and knew they were not alone in their concerns. This supportive environment was instrumental in preparing them for the demands of working independently:

Supporting each other, and when you’re so new, having each other’s backs during the first internship before going solo – it was a bit of a soft start. (Newly employed nurse, 3:81)

The clinical supervisors emphasized PL in preparing the newly employed nurses for independent practice. They highlighted that PL allows nurses to practice and learn from their mistakes within a supportive environment. While acknowledging the value of individual clinical training, they stressed that PL provided a stronger foundation for increased self-confidence built in a safe learning environment. Therefore, PL was perceived as a good start before working independently:

I don’t think we should remove the individual phase entirely, but peer learning builds a stronger foundation before they step out on their own. (Clinical supervisor, 6:135)

The newly employed nurses and clinical supervisors said that reflection and feedback were essential to this phase. Early opportunities to discuss the structure and purpose of PL with supervisors and peers clarified expectations at the beginning of the practical training.

#### Adapting to the ambulance nurse role through peer learning

PL was described as significantly enhancing role clarity for newly employed nurses, allowing them to adapt to and understand their responsibilities and work processes in a “real-world setting”. The newly employed nurses emphasized that the structure of PL mirrored the dynamics of ambulance work and what was expected of a nurse in the ambulance:

We [i.e., the peers] divided responsibilities beforehand. One of us would be the primary caregiver for a patient, asking most of the questions and taking charge of the patient’s care. The other would act as support, helping with tasks like taking vitals, speaking with family members, or packing up equipment. We tried not to interfere too much so the primary caregiver had the chance to do their job fully. A bit like you do in the ambulance when you sit in front like the experienced team. (Newly employed nurse, 8:28)

The clinical supervisors confirmed the newly employed nurses’ descriptions of role clarity and the possibility of adapting to the role they built using PL. They described how PL allowed the nurses to understand the workflow and role differentiation. Role clarity emerged naturally as peers worked together and communicated openly, making it easy for the clinical supervisor to support and follow their work. In addition, the clinical supervisors emphasized establishing roles and expectations early during the PL to ensure a productive and safe learning environment for the newly employed:

Peer learning ensures that nurses are not just observers but active participants who understand their responsibilities and role in the team. (Clinical supervisor, 6:109)

The newly employed nurses described how, even with the structured support provided by PL, a sense of pressure, expectations, and intense demands could engender struggle for them. PL was also discussed as a means of easing the transition, offering opportunities for collaborative problem-solving, mutual reflection, and knowledge-sharing with peers and clinical supervisors. The newly employed nurses described how PL enabled them to observe and engage in real-time decision-making, which helped them gradually build confidence in handling challenges in their ambulance work.

The newly employed nurses described how the unpredictability of patient conditions in the ambulance service added another layer of difficulty when adapting to their role. They compared their experiences with previous hospital experiences in which new nurses were gradually introduced to handling critical cases. In this new setting, they had to adapt immediately to caring for all patients, regardless of severity. The clinical supervisors acknowledged these challenges and emphasized to the newly employed nurses during their PL that adapting to a new role in a new setting requires more than clinical competence. All participants discussed and described how the PL provided a framework for discussing complex patient cases, reflecting on past experiences, and reinforcing critical thinking skills needed in unpredictable environments:

I definitely felt a lot of stress in situations in which the patients could be very seriously or life-threateningly ill. When you’re working with new employees, you can often choose patients who aren’t in such critical condition. Here, we didn’t get to choose our patients – it depended on the calls we received. So it was a more stressful situation. Some patients were less sick, while others were more critical, but it was still my job to assess and resolve the situation. (Newly employed nurse, 8:19)

The newly employed nurses described PL as providing support in adapting to the ambulance context. This reinforced the need for structured support and training to ease the transition into and adaptation to this demanding role:

Even if you have worked in healthcare for a long time, working in the ambulance is something completely different. (Newly employed nurse, 2:2)

A concern among the clinical supervisors, about adapting to the new role, was the establishment of workplace culture and professional behaviour. The clinical supervisors described how they strove to be role models and professionals, and to prevent the culture from negatively affecting the new employees during PL. They acknowledged that the supervisor’s role requires both professional competence and the ability to foster a supportive learning environment. This was possible under PL, as the clinical supervisors were always close to the new employees and could influence them professionally. They emphasized the importance of modelling positive behaviour, creating a welcoming atmosphere for the newly employed nurses during PL:

If we, as supervisors, describe all calls as terrible or say things like, ‘What kind of idiot called this in?’ – if that’s the constant tone – it establishes the culture we’re introducing the new colleagues to. They’re likely to adopt that behaviour quickly. (Clinical supervisor, 6:109)

### Safe space for shared learning

#### Collaborative teamwork

Teamwork between peers was fundamental to the PL experience, allowing the nurses to feel part of a solid unit. The newly employed nurses described how working closely with a peer helped them navigate challenges more effectively, as they could rely on each other for emotional and practical support. This collaborative teamwork fostered a sense of belonging and reassurance among the nurses, which was particularly important in the initial stages of their professional journey during the introductory training. They felt less overwhelmed and better equipped to handle complex situations by sharing responsibilities and supporting one another through teamwork during the PL. The peers in the collaborative team also played a crucial role in each other’s professional development by allowing knowledge and skills to be shared openly without rivalry. The absence of competition between the team members created a safe space for shared learning:

You don’t feel alone or like you’re the only one making mistakes, because there are two of you figuring things out together, and that’s very reassuring. (Newly employed nurse, 4:44)

The clinical supervisors highlighted the long-term value of teamwork fostered during the PL. They observed that these early partnerships within the collaborative team helped the nurses develop trust, independence, and teamwork skills. The clinical supervisor described developing team skills as crucial because, at some point, a clinical supervisor would not be part of the newly employed nurses’ team, and the teamwork needed to function in the presence of other colleagues:

At the same time, these are our new colleagues – people we’re introducing to teamwork – and I might end up working with them in just a month or so. (Clinical supervisor, 6:96)

Beyond collaboration with peers, PL also fostered personal and professional networks, which the nurses found equally valuable. These personal relationships often extended beyond the PL, creating a network of trust and shared understanding that could be drawn upon in future challenges. The newly employed nurses discussed this blend of professional and personal growth as underscoring the impact of PL: “We became very good friends through peer learning” (Newly employed nurse, 4:10).

The newly employed nurses appreciated discussing cases and exchanging feedback with their peers, which helped them evaluate their performance and identify areas for improvement. Furthermore, the clinical supervisors expressed the need for time for feedback and reflection after every patient encounter and at the end of the shift, before the newly employed nurses went home, noting that PL facilitated such time. Group feedback was a way for the nurses to reflect on and provide feedback on their team’s performance. Still, the clinical supervisors also emphasized the importance of individual feedback, as the nurses might be at different levels, and the clinical supervisor needed to help each nurse develop in their professional role:

Many are at slightly different levels. Therefore, I think feedback needs to be individualized to help each person develop as an individual, not just as part of a group. (Clinical supervisor, 5:25)

#### Overcoming fears in peer learning dynamics

While PL offers many benefits, the newly employed nurses and the clinical supervisors highlighted its potential downsides, particularly regarding group dynamics. The newly employed nurses expressed concerns that team dynamics could negatively affect their learning experiences during PL. Some initial concerns at the beginning of their PL were that their peers would be more skilled and would not benefit from working with them, or, conversely, that their peers would not give or receive the same amount of support. The nurses with no previous experience in PL feared that they would be in the way or not be seen as equals:

I mean, I think the risk of peer learning could be stepping on each other’s toes – that’s the potential downside I can see. (Newly employed nurse, 4:9)

For some, PL created uncertainty, particularly when nurses felt they could not rely entirely on their peers. In some instances, the pressure to collaborate with a peer who had a different approach or skill level led to feelings of self-doubt and created an imbalance in confidence within the pair:

While I supported her with her patients, I didn’t always feel I could get the same support back from my peer. (Newly employed nurse, 4:39)

The clinical supervisors acknowledged PL’s challenges, particularly when the newly employed nurses struggled to navigate the team and their peers. The clinical supervisors noted that while PL fostered collaboration, it could also create tension or competition if not managed effectively. The clinical supervisors needed to adapt their approach to address the peers’ individual challenges and be sensitive to tensions or conflicts within the peer team. The clinical supervisors noted that, in most cases, the tension stemmed from uncertainty among one of the newly employed nurses on the peer team. When that nurse’s peer had different experiences and a different level of self-confidence, this could cause conflicts, and the clinical supervisor then needed to step in and be active in the peer team’s supervision. For example, if one peer took over too much and did not allow their peer opportunities to participate in patient care, the clinical supervisor needed to help create space for the other peer. When the peer team did not cooperate and function well, more time and experience were required of the supervisor to handle the situation, and this was considered challenging for the clinical supervisors and their supervisor colleagues:

I’ve had teams that worked well and teams that didn’t. Those situations have been challenging for me as a supervisor and for my colleague as well. (Clinical supervisor, 6:69)

#### The shared journey of peer learning

The newly employed nurses highlighted the value of shared experiences in developing mutual understanding and confidence during their onboarding process and PL. They described how working alongside their peers allowed them to relate to one another’s challenges and progress. This shared journey was seen as a source of comfort and reassurance, creating an environment where learning felt collaborative and less isolating. Knowing that one’s peer was facing similar obstacles helped reduce anxiety and encouraged open discussions of both successes and difficulties. The camaraderie developed through PL also enhanced the nurses’ motivation and engagement in their work. The newly employed nurses felt supported and understood when working with a peer who could empathize with their experiences. Their shared experiences allowed the nurses to bond over common goals and challenges, fostering a sense of belonging and community within the learning environment during their PL:

That case took quite a lot out of us, I remember. We got out of the car and [note: sighed loudly] and had to breathe. And then it was so nice that it was the same for both of us. … And we had the same feelings afterward and could discuss [things], and you understood each other in a different way than with someone who had not been at the scene. (Newly employed nurse, 2:18)

The clinical supervisors noted that the newly employed nurses gained comfort by being together in a peer team. The clinical supervisors also reflected on their own experiences of being new and the feelings that accompanied this. Opportunities for mutual reflection fostered a shared emotional and professional narrative, strengthening a sense of collective learning.

#### Diverse healthcare experiences enriched peer learning

The newly employed nurses described the diversity of professional backgrounds and prior experiences among their peers as a significant strength of the PL process. The nurses discussed how their different areas of expertise and knowledge allowed them to complement each other’s strengths and address knowledge gaps, allowing them to gain insights from their peers and different perspectives from each other:

We were able to share our knowledge. She had worked in many different hospital wards, and I had worked a lot in the emergency department, so it felt like we complemented each other well and learned from each other. (Newly employed nurse, 4:5)

Such knowledge exchange was particularly valuable in challenging patient care situations when a newly employed nurse had relevant expertise that could directly enhance care. Patient care benefited from the team members’ different experiences, as they cared for the patient as a team:

Once, we visited a haematology patient, and I had no idea how to handle the situation. However, my peer had it completely under control because she had worked with that type of patient before. This resulted in much calmer and more efficient management of the patient because she knew what to expect from that type of case. (Newly employed nurse, 4:7)

The clinical supervisors observed that these diverse peer experiences promoted adaptability and broadened the newly employed nurses’ understanding of the new clinical context. Sharing knowledge and techniques from different cases also enhanced critical thinking and the ability to apply their theoretical knowledge to solve practical challenges. The complementary nature of the peer team’s skills improved patient outcomes and fostered a dynamic, supportive learning environment. This shared learning reinforced the value of diverse professional experiences during PL:

So far, it feels like they’ve been able to help each other. Even if one had more experience, they’ve generally been good at letting each other take the lead. (Clinical supervisor, 6:24)

### A balancing act as a supervisor

#### Fostering independence and confidence

The clinical supervisors played a central role in promoting independence and confidence among the newly employed nurses. With its structured approach, PL enabled nurses to gradually take on responsibilities, building their confidence step by step. This gradual exposure to independence was key to the nurses’ development of critical thinking skills and to their finding their new professional role. The clinical supervisors carefully balanced their involvement in the newly employed nurses’ learning by ensuring they felt supported while still encouraging them to step out of their comfort zones. The newly employed nurses expressed appreciation for PL, highlighting how it enabled clinical supervisors to create opportunities for them to lead the work while remaining available for guidance when needed. For example, the clinical supervisor guided them through the first patient interactions on day one and then gradually handed over responsibilities to the peer team on the first day of PL:

The supervisor handled the first two patients, but after that, I was the one taking the lead while they watched and guided me when needed. (Newly employed nurse, 4:16)

The clinical supervisors emphasized the dynamic nature of their role, balancing guidance and autonomy to foster independence among the newly employed nurses on the team. They described their role as providing support while encouraging the nurses to take the lead, stepping in only when necessary for patient safety, and highlighting the value of allowing the peer team to handle tasks collaboratively before they interacted in the care. The clinical supervisors described adapting their level of involvement to the situation, stepping back to let the nurses take the lead when appropriate, and remaining available to intervene if necessary to ensure patient safety. This adaptability was discussed as central to fostering trust and a sense of security among the newly employed nurses while maintaining patient safety. The newly employed nurses appreciated supervisors who provided a balance between offering support and allowing them to take responsibility. This approach helped them feel confident in their abilities while knowing that help was always available if needed:

The supervisor did it in a good way. It wasn’t like the supervisor just walked in and took over, and we weren’t allowed to be involved. I feel that peer learning is like that. We have support behind us without someone taking over, but guiding us in the right direction. (Newly employed nurse, 2:20)

The clinical supervisors highlighted the importance of allowing nurses to learn through hands-on experience, even when minor mistakes occurred. With critically ill patients, when the clinical supervisor had to step in and take the lead, there were other learning opportunities. For example, the clinical supervisor instructed the newly employed nurses to observe or delegated tasks for them to do:

To ensure that the right actions are taken, it doesn’t mean that the person being trained has to step back entirely. You can still delegate tasks to them. Afterward, we can hand it back to the pair, and as a supervisor, I can step back again. (Clinical supervisor, 6:28)

Supervised debriefings further helped the nurses reflect on their decisions and reinforced their learning, transforming experiences into actionable growth opportunities. During PL, clinical supervisors actively created debriefing and feedback spaces following patient encounters, using reflective dialogue to help newly employed nurses develop situational awareness and decision-making skills. According to the nurses, reflection and feedback contributed significantly to emotional readiness, as they felt reassured by the chance to process their experiences with their peers and clinical supervisors.

#### Supervisors providing accessible guidance

The newly employed nurses emphasized that peer support and supervisory guidance during PL created a safe space for asking questions and acquiring new skills. The nurses repeatedly underscored the importance of having approachable clinical supervisors available to provide answers during care. This PL environment allowed nurses to develop their skills without fear of judgment, making them feel safe and enhancing their learning experience. The newly employed nurses highlighted how their supervisors’ willingness to offer guidance during PL fostered a positive learning environment and boosted their confidence:

They were so good at teaching, and you could feel that you could ask any ‘stupid’ questions without being judged. They answered as best they could, and you could feel that they genuinely wanted you to succeed. (Newly employed nurse, 3:36)

The newly employed nurses described how, during PL, the clinical supervisors were more engaged and educational than the clinical supervisors during the individual training. During PL, the clinical supervisors recognized the importance of creating a supportive environment where nurses felt comfortable asking questions and making mistakes. These clinical supervisors highlighted the impact of their guidance in technical skills and in learning about the organization; moreover, they helped the nurses integrate into the workplace, as the ambulance context was described as differing drastically from the nurses’ previous workplaces:

It’s more about supporting them in what they are doing, as the other supervisor has said. That’s why the dynamic is so important, and as a supervisor, you need to be engaged the whole time. (Clinical supervisor, 6:28)

## Discussion

PL, introduced as structured learning within the introductory training programme, became a central component of the onboarding and learning process for newly employed nurses in the ambulance service. While PL was initially designed to support learning, the findings suggest a broader understanding of PL in this context. Based on the findings of this study, PL can be understood as a collaborative workplace learning process in which newly employed nurses shared responsibility for patient care and supported each other through feedback, validation, and joint reflection in unpredictable clinical situations. Clinical supervisors supported this process primarily by facilitating learning and ensuring patient safety.

The findings indicate that learning during the introductory training occurred through several interacting mechanisms. Observation, guided feedback from clinical supervisors, and peer interaction all contributed to professional development. However, PL appeared to function as the central organising structure during the clinical phase, as learning was actively negotiated between peers through shared responsibility, dialogue, and reflection rather than occurring primarily through observation alone. Participants described how PL helped clarify roles and responsibilities and supported active participation in clinical tasks. Instead of entering the workplace as passive observers, the nurses highlighted how PL promoted interaction, knowledge sharing, and collaborative problem-solving with peers and clinical supervisors. Clinical supervisors likewise described how PL made supervision more focused and purposeful.

Although PL proved valuable within the context of this study, it remains unclear whether the observed benefits were inherent to PL itself or were mainly due to the structured and standardised nature of the onboarding process. Nonetheless, the absence of a previously formalised introductory training programme underscores the importance of providing clear and supportive learning conditions for newly employed nurses.

An important aspect of PL was the opportunity to learn from peers’ diverse experiences and perspectives. Babinská and Pleschová [33] demonstrate that PL is strengthened when learners contribute different forms of experiential and contextual knowledge, enabling comparison of approaches and broadening clinical understanding. In the present study, such diversity appeared to support joint reasoning and shared sense-making in complex clinical situations. These findings align with previous research showing that PL interventions during workplace introductions increase new graduates’ confidence and create safer and more interactive learning environments [17]. PL has also been shown to enhance clinical reasoning, critical thinking, and readiness for independent practice [17, 42].

A key factor contributing to PL’s effectiveness was the sense of psychological safety and peer trust reported by participants. In high-stakes environments such as ambulance services, newly employed nurses often face rapid transitions, complex patient situations, and limited opportunities for reflection. Being paired with a peer provided emotional support and created space for open dialogue, enabling nurses to share responsibility and manage challenging situations together. Peer collaboration, therefore, functioned not only as a learning strategy but also as a buffer against isolation and a source of validation in unfamiliar and demanding clinical settings. This finding reflects earlier research identifying peer support as an important resilience resource in emergency and acute care contexts characterised by rapid decision-making and emotional strain [17, 42, 43].

Clinical supervisors were also identified as pivotal to PL’s success. In this study, the clinical supervisors reflected on how using the PL changed their supervisory role, encouraging more structured engagement, ongoing observation, and approaches adapted to group dynamics and individual needs.

The clinical supervisors emphasised that PL allowed them to be more proactive in their teaching rather than only stepping in when problems arose. They also described how guiding two learners simultaneously required greater attentiveness but also led to deeper pedagogical reflection. Structured feedback and facilitated reflection were central tools in this process. The clinical supervisors’ reflections on their role resonate with research emphasizing the importance of reflective and dialogic conversations for meaningful professional development. Pleschová et al. [44] describes how sustained, trust-based conversations between educators and learners can lead to deeper reflection, shifts in understanding, and long-term change in professional practice. In the present study, structured feedback and facilitated reflection within the PL appear to function as such meaning-making conversations, supporting not only skill acquisition but also the development of professional identity among newly employed nurses. This is supported by previous research showing that timely, individualized supervision contributes to role clarity, confidence, and overall professional development among newly employed nurses. The clinical supervisors reflected on their influence as educators and as carriers of workplace culture. They were aware that the attitudes and behaviours they demonstrated in everyday interactions served as models for the newly employed, contributing to the internalization of professional norms and values. This study revealed how clinical supervisors balanced supporting learning with being mindful of their responsibility to model professionalism, communication, and respect. These elements position clinical supervision not only as a pedagogical task but also as a tool for socialization into the workplace [17].

These findings are consistent with Bandura’s social cognitive theory [28], which highlights the importance of observational learning and the role of modelling in shaping behaviour and internalising norms. Individuals learn not only through direct experience but also by observing others’ actions and their consequences. In the PL context, clinical supervisors serve as role models whose behaviours, values, and interactions become central learning cues for newly employed nurses [28]. While observational learning formed an important component of clinical learning, particularly in high-risk situations requiring supervisor intervention, observation alone did not appear to drive learning. Instead, observations were typically followed by peer discussion and facilitated reflection, suggesting that meaning-making occurred primarily through PL processes.

This study describes feedback and reflection as central and recurring aspects of the learning process for newly employed nurses during PL in the ambulance service. Feedback during PL is depicted as a source of reassurance, helping nurses navigate the uncertainty of a new, often unpredictable care environment. PL provides a safe learning atmosphere and encourages reflective feedback from peers and clinical supervisors. This aligns with the findings of Pålsson et al. [17], who reported that a PL intervention during the workplace introduction of newly graduated nurses led to increased professional confidence and smoother integration when the organization and clinical supervisors actively supported feedback and collaboration [17]. However, while feedback and reflection are widely recognized as essential components of clinical supervision, their consistent application across healthcare settings remains a challenge. In many traditional supervision models, the lack of structured opportunities for feedback and reflection can hinder learning. This inconsistency may stem from limited supervisor training, a lack of structured frameworks, or varying motivation levels among clinical supervisors. In contrast, the PL highlights collaborative learning and mutual support, fostering an environment where feedback and reflection are integral to daily practice. The psychological safety inherent in PL environments encourages open communication, allowing nurses to share experiences and insights without fear of judgment. This supportive atmosphere enhances individual learning and builds a cohesive team dynamic. Studies have shown that environments with clear structures, dedicated supervision, and psychological safety improve clinical learning outcomes and professional confidence among nursing students and newly employed nurses [45, 46]. To bridge the gap in traditional supervision models, it is essential to incorporate structured training programmes that emphasize feedback and reflection. Clinical supervisors can create more effective and supportive learning environments by adopting elements of the PL, such as collaborative learning structures and fostering psychological safety. This approach benefits newly employed nurses and may enhance the overall quality of patient care through the ongoing professional development of their carers.

Regardless, according to Pålson et al. [17], both managers and newly graduated nurses emphasized that structured and guided PL, underpinned by feedback, facilitated socialization into the professional role and enhanced learning outcomes; feedback was a pedagogical tool and a strategy for ensuring patient safety and fostering mutual trust [17]. Ericsson et al. [43] underlined that regular, constructive feedback from colleagues and clinical supervisors is a key resilience resource for newly employed professionals in demanding environments such as ambulance services. These previous findings and our indicate that feedback improves clinical performance and emotional security, especially when professionals are expected to assume responsibility early in their careers. In contrast, the absence of clear or consistent feedback can undermine confidence and delay adaptation [43]. Studies also show that effective feedback is associated with the development of clinical reasoning, improved team collaboration, and enhanced patient care outcomes [47].

In summary, integrating PL into the introductory training of newly employed nurses in the ambulance service appears to provide a structured and effective way of supporting their transition into practice. It is strengthened by collegial trust and supported by engaged clinical supervision, which together offer a robust framework for professional development for newly employed nurses. Together, these components enhance learning, provide emotional and practical support, and foster a culture of reflection and professional growth. In complex clinical environments such as ambulance services, where autonomy, adaptability, and resilience are essential, PL may contribute to sustainable onboarding and retention by supporting collaborative learning and professional development.

### Strengths and limitations of the work

Two authors, KJ and VL, collected the data, maintaining a consistent approach through continuous discussions before and after the interviews. However, the data collection also had limitations, as it was confined to one ambulance organization contracted by the Stockholm region. Despite Sweden’s formal regional framework, the introductory training may differ across organizations and regions, potentially affecting the transferability of the findings. The study aimed to describe the use of peer learning (PL) and how it was experienced during the introductory training of newly employed nurses in a regional ambulance service. However, the worldwide similarity in ambulance services in providing care for accident victims and those with acute illnesses, irrespective of predetermined care environments [48], supports the assumption that our findings could apply to other ambulance service organizations.

Regarding demographics, nine out of ten nurses were women. This homogeneity may have limited data diversity. However, given the trend of rising female employment in ambulance services [49], the participants likely accurately reflect the current workforce composition.

Three authors, two female and one male, had substantial experience working as SNs in the ambulance service, which may have introduced bias into the study design, data collection, and analysis. To minimize bias, the analysis process included critical discussions. While potential biases do exist, the authors’ preconceptions also contributed positively by enhancing their ability to interpret and understand the study findings [50]. These authors’ backgrounds in and experiences of the study context likely influenced the interpretation of the collected data.

### Implications for policy and practice

This study highlights the value of PL as part of introductory training in ambulance services. The findings suggest that PL can support newly employed nurses’ transition into autonomous clinical roles by fostering collaboration, reflection, and shared responsibility for patient care. To support the use of PL in introductory training, the organisations need to provide preparation for clinical supervisors, allocate time for feedback and reflection during clinical shifts, and incorporate peer learning activities into existing onboarding programmes. PL may represent one way to strengthen introductory training for newly employed nurses in demanding clinical environments. Providing structured opportunities for peer interaction and supervised reflection may help support professional development, teamwork, and confidence during the transition into ambulance service.

## Conclusions

This study shows that PL represents a valuable learning phase within the introductory training programme for newly employed nurses in the ambulance service. PL contributed to the development of clinical competence, emotional readiness, and professional confidence through shared responsibility, reflection, and active collaboration among peers, while clinical supervisors played an essential facilitating role in ensuring patient safety and guiding learning.

Learning occurred through several interacting mechanisms, including peer support, supervisor guidance, and observation in complex clinical situations. However, PL appeared to structure how experiences were reflected upon and transformed into professional learning. These findings suggest that integrating PL into introductory training programmes may support a safe, sustainable transition into practice in challenging, unpredictable clinical environments.

## Relevance to clinical practice

In complex and time-sensitive care settings such as ambulance services, newly employed nurses often face immediate demands for independence, adaptability, and clinical judgement. This study demonstrates that PL fosters a supportive, collaborative learning environment that enhances skill acquisition, reduces stress, and builds confidence. Through shared experiences and supervisor-guided reflection, nurses can integrate into the clinical team and better manage the unpredictability of care in the ambulance service. Implementing PL in ambulance services can improve onboarding quality, strengthen team dynamics, and ultimately contribute to better patient care outcomes.

## Data Availability

The datasets used and/or analysed during the study are available from the corresponding author upon reasonable request.
